# All who wander are not lost: the search for homology during homologous recombination

**DOI:** 10.1042/BST20230705

**Published:** 2024-02-07

**Authors:** Jingyi Hu, J. Brooks Crickard

**Affiliations:** Department of Molecular Biology and Genetics, Cornell University, Ithaca, NY 14853, U.S.A.

**Keywords:** DNA repair, helicases, homologous recombination, homology search, Rad51

## Abstract

Homologous recombination (HR) is a template-based DNA double-strand break repair pathway that functions to maintain genomic integrity. A vital component of the HR reaction is the identification of template DNA to be used during repair. This occurs through a mechanism known as the homology search. The homology search occurs in two steps: a collision step in which two pieces of DNA are forced to collide and a selection step that results in homologous pairing between matching DNA sequences. Selection of a homologous template is facilitated by recombinases of the RecA/Rad51 family of proteins in cooperation with helicases, translocases, and topoisomerases that determine the overall fidelity of the match. This menagerie of molecular machines acts to regulate critical intermediates during the homology search. These intermediates include recombinase filaments that probe for short stretches of homology and early strand invasion intermediates in the form of displacement loops (D-loops) that stabilize paired DNA. Here, we will discuss recent advances in understanding how these specific intermediates are regulated on the molecular level during the HR reaction. We will also discuss how the stability of these intermediates influences the ultimate outcomes of the HR reaction. Finally, we will discuss recent physiological models developed to explain how the homology search protects the genome.

## Introduction

**Figure 1.**
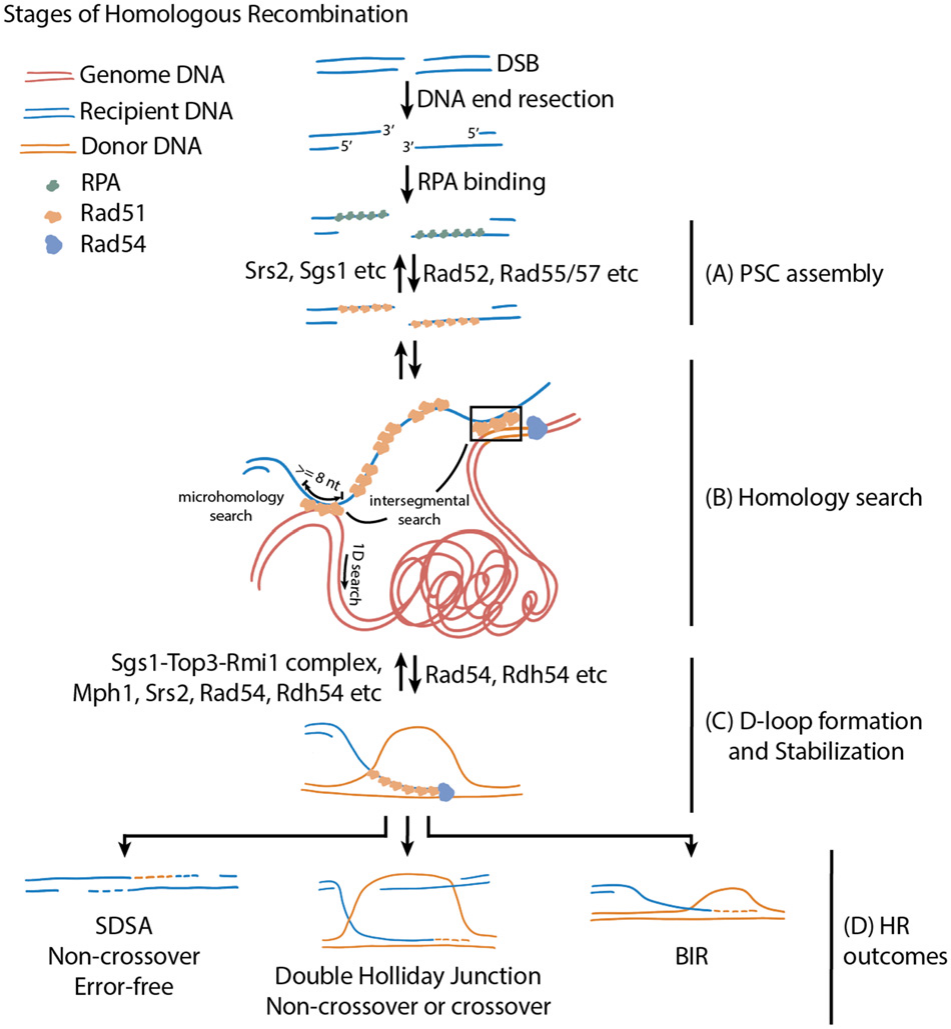
Illustrating the HR process in *Saccharomyces cerevisiae*. Homologous recombination (HR) initiates with DNA end resection of a DSB, creating 3′ overhangs of ssDNA. The ssDNA is initially bound by replication protein A (RPA) and (**A**) followed by recombinase binding which is regulated by a series of mediators and anti-recombinases, culminating in the formation of an active presynaptic complex (PSC). The PSC gains the capability to perform (**B**) the homology search among the genome by microhomology kinetic search, intersegmental search, and local 1D sliding search. Once the homologous sequence is identified, the ssDNA invades the dsDNA to form a D-loop. (**C**) Zoom in the black box in (**B**). The D-loop is reversible and regulated by several helicases and a topoisomerase, which eventually leads to different (**D**) HR outcomes.

DNA double-strand breaks (DSBs) are a dangerous type of genomic lesion that can rapidly degrade information stored within chromosomes. Homologous recombination (HR) is a template based DSB repair pathway that maintains the integrity of chromosomes in all organisms. Critical during both phases of the eukaryotic life cycle, HR protects genomic integrity during mitotic growth and promotes genetic diversity during meiosis [1,2]. HR can start with the formation of a DNA break. After the break is identified, the DNA is resected from the 5′ to 3′ direction to create ssDNA that can then be used as a guide by recombinase filaments to find a matching sequence elsewhere in the genome [3–5] (Figure 1). The key recombinases are RecA in eubacteria, RadA in archaea, and Rad51/Dmc1 in eukaryotes [6,7]. Dmc1 is meiosis specific [8].

The homology search step controls template selection by pairing the damaged recipient DNA to a matching donor DNA [9]. The identity of this donor then governs the fidelity of the reaction. In most HR reactions, the sister chromatid is used to repair the DNA [10]. This is considered the highest fidelity repair pathway. Changes to the genome can occur when an ectopic sequence or a homologous chromosome is selected, leading to potential gene conversion and crossover (CO) outcomes [11–13]. In the case of meiotic HR, gene conversion and COs are used to promote genetic diversity within populations. Therefore, mechanisms of template selection are deeply entrenched in genome maintenance, both on a short-term and a long-term timeline. This review will focus on the homology search and template selection during mitotic growth. While conceptually similar, mechanisms that govern meiotic homology search involve a different set of genes. We direct you to the following references [14,15] for a review on this topic.

## RecA/Rad51 family recombinase, the engine that drives the homology search

Recombinase proteins are conserved across the kingdoms of life [6]. The general role of the RecA/Rad51 family of recombinases is to pair donor and recipient DNA. RecA/Rad51, a family of DNA recombinases, catalyzes the homology search during HR by assembling as a filament on ssDNA called the presynaptic complex (PSC). The PSC can interrogate the genome for a matching sequence element in the donor dsDNA. Once the specific homology has been identified, the filament transitions into a strand invasion reaction. In this reaction, the ssDNA anneals with the complementary strand and displaces the non-complementary strand to form a displacement loop (D-loop).

The filamentous structure of recombinases is one of the defining features of this group of enzymes. They form a right-handed helical filament with a ∼95-Å pitch and a turn of ∼6 RecA monomers on DNA. This structure stretches the DNA 1.5 times, lengthening the DNA [14–16]. Although the DNA is stretched by RecA filaments globally, it is locally organized into B-DNA-like triplets, allowing them to recover energy through base stacking [15]. Physical modeling suggests that the binding and opening of a dsDNA involves the secondary DNA binding site and the C-terminal domain (CTD) of the RecA filament [17,18]. Cryo-electron microscopy demonstrated that upon binding to the CTD, dsDNA is directed and opened by the RecA L2 loop. This facilitates the subsequent sequestering of the non-complementary strand to the secondary DNA binding site. This promotes the pairing between the recipient and donor DNA [16,19]. The opening of dsDNA can be terminated at each protomer step, with the homologous DNA sequence suppressing the termination. This mechanism enhances the fidelity of the homology search [16,20,21].

The direct visualization of reconstituted systems has defined the assembly of both RecA and Rad51 on ssDNA. Filament assembly occurs by a two-step kinetic mechanism where RecA nucleates on transiently exposed ssDNA followed by extension and filament growth [22]. Similar observations have been made for Rad51 [23]. Fluorescence microscopy has also revealed a direct mechanism by which the RecA/Rad51 family of proteins reduces the complexity of the homology search by adopting a length-based microhomology recognition. Recombinase filaments kinetically reject dsDNA lacking 8-nt microhomology motifs [24–26]. The PSC can also probe different dsDNA regions simultaneously by ‘intersegmental contact sampling’ in a three-dimensional manner to increase the probability of encountering the target and decreasing reaction time [16,27]. The biophysical properties of RecA/Rad51 filaments have generated a wealth of knowledge that can be used to interpret recent advances in *in vivo* imaging used to track recombinase activity, as discussed below.

## Versatile factors help with the homology search

Without an active recombinase filament, the homology search will not go to completion. Therefore, we consider filament assembly and dynamics to be key regulators of the homology search (Figure 1A). This section will discuss some of the many eukaryotic mediator proteins that ensure proper assembly and regulation of Rad51 filaments during the homology search.

The formation of fully active Rad51 filaments in eukaryotes is dependent on mediator proteins, including Rad52 [28,29] in *Saccharomyces cerevisiae* and BRCA2 (Breast cancer type 2 susceptibility protein) [30–33] in humans. Several Rad51 paralogs, including Rad55/57 [34] and Csm2-Psy3-Shu1-Shu2 (known as Shu complex) [35] in yeast and RAD51B, RAD51C, RAD51D, XRCC2, XRCC3 [36–38] in higher eukaryotes also regulate RAD51 filaments. The necessity of these mediators occurs due to differences between RecA and Rad51. RecA inherently differentiates between binding to ssDNA and dsDNA, while Rad51 requires additional factors to promote productive binding to ssDNA over dsDNA [39]. Rad51 also competes with replication protein A (RPA), which binds to resected ssDNA following DSBs, preventing the formation of secondary structures [39]. The Rad51 mediators relieve these competitive inhibitors for ssDNA binding. Examples of these mechanisms include; BRCA2 nucleating RAD51 binding events along the DNA [40,41], a role carried out by Rad52 in *S. cerevisiae* [42], Rad51 paralogs Rad55/57 serving as mediators to regulate the assembly and reassembly of Rad51 filaments [43,44], and RAD51B-RAD51C-RAD51D-XRCC2 complex (BCDX2) stimulating the nucleation and extension of RAD51 filaments [40,45].

Rad51 also recruits the DNA motor proteins Rad54 and Rdh54, known as RAD54L and RAD54B in higher eukaryotes, during the homology search and strand invasion steps. These two motor proteins promote D-loop formation [46–57] and translocate along dsDNA to support a chromosome-remodeling activity, which aids recombination in the context of chromatin *in vivo* [50,58,59]. Importantly, they prevent toxic accumulation of Rad51 on dsDNA, which is important for maintaining Rad51 pools necessary for promoting productive recombination [60–62]. Additional helicases also contribute to balancing Rad51 pools by controlling Rad51 filaments bound to ssDNA. These include Srs2, Sgs1, and Mph1 in *S. cerevisiae*. Srs2 functions by stimulating the ATP hydrolysis activity of Rad51, causing dissociation from the ssDNA [63–66]. The anti-recombinase activity of Srs2 can be restricted by cooperative interaction between motor proteins Rad54 and Rdh54 [67]. The activity of the Rad55/57 complex also antagonizes Srs2 by forming a more complete Rad51 filament [43,68]. Factors that load Rad51 on ssDNA and modulate the activity of the filament collaborate to balance recombinase pools required to maintain healthy recombinase filaments through both direct and indirect means.

## The reversibility of Mitotic recombination intermediates

The initial sequence selection of homologous DNA is kinetic (Figure 1B). However, correct sequence identification requires the maturation and stabilization of D-loop structures. This makes D-loop stability an inherent regulator of the homology search. This section will focus on how D-loop regulation factors into the homology search pathway (Figure 1C) and exit from the homology search step in HR.

Several helicases can disrupt the nascent D-loop. For example, Sgs1-Top3-Rmi1 (known as STR complex) in yeast, and their human homologs BLM (Bloom syndrome protein) -TOPO3α-RMI1-RMI2 complex, Mph1 in yeast known as FANCM in mammals, and Srs2 in yeast. The homolog of Srs2 in mammals is still unclear but is thought to be FBH1, a UvrD helicase [65,69]. Each of these motors promotes mechanistically distinct modes of D-loop regulation. Biochemical studies have shown that Sgs1 alone can dissociate protein-free D-loops but cannot dissociate D-loops formed with Rad51, Rad54, and RPA proteins. In this situation adding Top3-Rmi1 dissolves D-loops in a topoisomerase-dependent manner [70]. Human BLM is less efficient in disrupting D-loops unless complexed with TOPO3α-RMI1-RMI2 [71]. Mph1 can disrupt both protein-free D-loops and those containing Rad51, Rad54, and RPA [65]. Finally, Srs2 can only disrupt D-loops composed of Rad51 and is kinetically blocked by the cooperative efforts of Rad54 and Rdh54 [67]. These differences imply that each helicase may function at unique stages of nascent D-loop metabolism.

Recent work in *S. cerevisiae* has identified two types of nascent D-loops regulated by specific helicases. Srs2 controls one type, and the STR complex, along with Mph1, modulates the other. Rdh54 helps delineate these two pathways and shifts the process toward the STR-Mph1 axis [54]. These pathways may also be delineated by the enzymatic activities of their regulating proteins. For example, Top3 is a type 1A topoisomerase that can introduce positive supercoils into DNA, which could contribute to destabilizing D-loop structures [70,72]. In contrast, Srs2 strips Rad51 filaments from DNA and disrupts short D-loops that may form in the presence of Rad51 alone [63,69]. A fundamental question about D-loop reversal post-synapsis is the stability of Rad51 filaments. Two primary models exist. One model predicts continuous disruption of Rad51 filaments at D-loops, requiring cycles of filament disruption and re-formation to identify the homologous sequence. Alternatively, Rad51 filaments may remain stable during the homology search, and reversal mechanisms may rely on strategies that manipulate the donor DNA and do not cause disruption of Rad51 filaments. Versions of both models likely contribute to the regulation of nascent D-loops. Further work will need to be done to understand the difference between these types of D-loops and a definite reversal mechanism.

The stability of mature D-loops also relies on the length of homologous pairing. Longer tracts of invading homology will become stable intermediates [59] whose stability depends on the sum of the stability of all the bases in the product [73,74]. Regulation of stable intermediates is then some combination of helicase activity and the efficiency of base pairing within the DNA joint molecule. For example, if many mismatches are present, reversal is more likely with weaker helicase activity. In contrast, if the sequence is highly complementary, strong helicase activity will be required for disruption. This activity is also regulated by mismatch repair proteins, which can be reviewed here [75].

D-loop disruption is also a critical component of the synthesis-dependent strand annealing pathway. This occurs after D-loop extension (Figure 1D). In this pathway, the newly synthesized DNA strand anneals with the second broken end. This promotes DNA replication of the second end using the newly synthesized strand as a template [76–78]. If the D-loop is not disrupted, the extended D-loop gains the ability to form a double Holliday junction (dHJ) structure. Regulation of dHJ necessitates structure-selective endonucleases that recognize and cleave DNA junctions. Resolution of recombination intermediates will finally go to either CO or non-CO outcomes [79,80]. If no second end is found, long-range DNA replication can occur as part of the break-induced replication (BIR) pathway [81,82]. Both CO and BIR pathways can lead to loss of heterozygosity if the template is an ectopic sequence or the homologous chromosome [83].

Mechanisms underlying the dynamic regulation of PSCs and D-loops are fundamental to defining how gene conversion and CO events can occur. Deletion of any one of the helicases mentioned above causes an increase in CO [65,84–86], suggesting a more stable intermediate may be more prone to CO events during mitotic HR. Future work will need to better define the process of template selection and HR outcomes.

## Physiological mechanisms of homology search

We have discussed the molecular underpinnings of DNA selection during the homology search. However, for selection to occur, DNA from different sites in the genome must physically meet each other. This requires the large-scale movement of chromosomes to increase contact probability between recipient and donor sites. While there are shared features between eubacteria and eukaryotes, there are also some major differences. This section will discuss models for the *in vivo* homology search.

In eukaryotes, chromatin has a significant role during the homology search. Activities associated with chromatin include managing the DNA damage response signaling pathway [87,88], chromosome movement [89], topological domain organization [87,90], and proper stabilization of strand pairing [91]. These roles are too substantial to productively review here, and instead, we direct the reader to some of the excellent papers listed above.

The net result of chromatin remodeling in response to a DSB is increased diffusive movement of chromosomes within the nucleus, which leads to productive collisions between donor and recipient DNA molecules [89,92]. In diploid yeast, this movement can promote recombination events between homologous chromosomes [92]. More recent evidence suggests that a global reduction in nucleosomes may also improve the homology search by decompaction of localized domains, creating more efficient searches [93,94]. A second component of chromosome movement during the homology search is the recruitment of the Arp2/3 complex to sites of DNA DSBs [87,95–98]. The polymerization of nuclear actin is a requirement for chromosome movement, promoting improved collisions between distal sites within the genome [92]. Together, this response improves collisions between DNA sites within the genome.

The structural maintenance of chromosome (SMC) family of proteins is important during the homology search in both prokaryotes and eukaryotes, although these activities need further definition. This group of proteins includes the Condensin, COHESIN, SMC5/6 proteins, and RecN in prokaryotes [99]. Recently, it has been shown that COHESIN acts as a topological barrier to promote local homology search and inhibit long-range homology searches that can lead to genomic rearrangements [90,100]. While the structural contribution of these proteins has been identified, more work will be needed to understand how their role in the topological insulation of specific domains may contribute to the rates of homologous DNA selection.

Together, the chromatin structure in the form of nucleosomes, SMC proteins, and the nuclear cytoskeleton collaborate to improve chromosome movement during the homology search. This leads to an increased donor DNA identification. Future work will need to understand how movement is connected to the DNA template selection process.

## Current cytological models for the homology search

**Figure 2.**
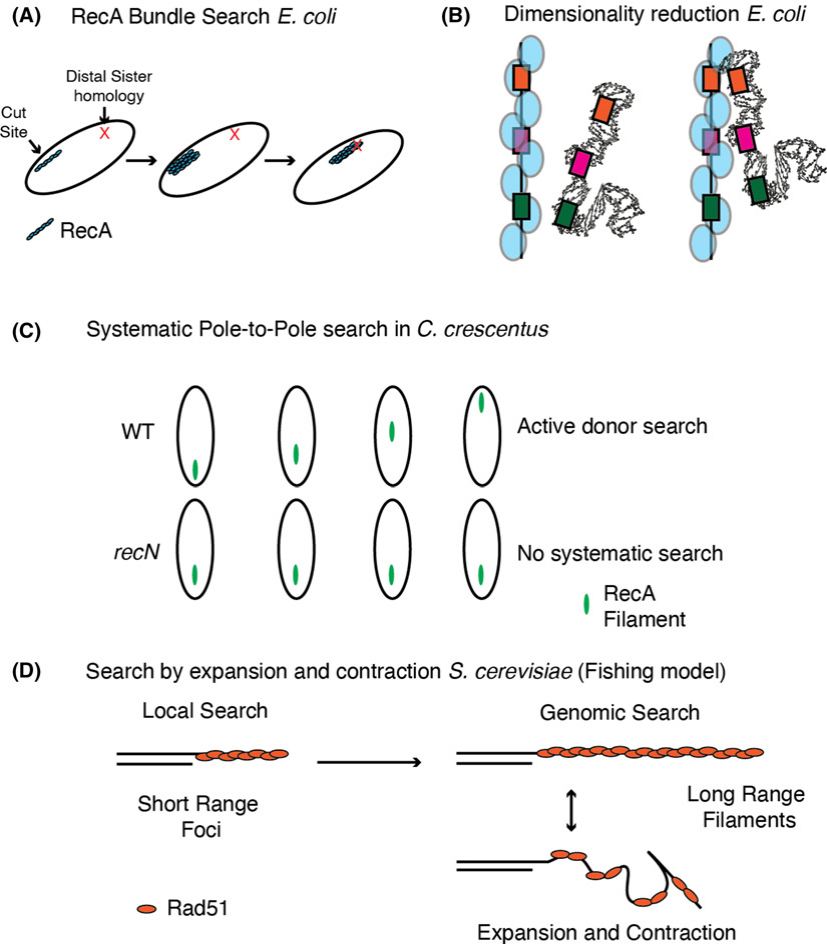
Cytological models of the homology search. (**A**) The RecA bundle model proposes that RecA nucleates at sites of double-strand breaks and then forms a bundle of filaments to promote the homology search. This allows the identification of distant sister sites. (**B**) The dimensionality reduction model proposes that the growth of the RecA filament allows a matching sequence of the donor strand to dimensionally match at least a small portion of the RecA filament, which can then recognize a matching sequence in 2D space. (**C**) The direct pole-to-pole movement of RecA filaments observed in *C. crescentus*. Movement is dependent on the SMC motor protein RecN. (**D**) An expansion and contraction model were developed from *in vivo* imaging of Rad51. This model proposes that rounds of expansion and contraction help to promote pairing during the homology search.

Direct labeling of RecA generally results in the inactivation of the enzyme, which makes tracking the protein *in vivo* challenging. However, several strategies have been employed to solve this problem. These experiments have developed cytological models and provided a general understanding of the RecA/Rad51-mediated homology search in cells. In this section, we will discuss these models (Figure 2).

A standard method for visualizing RecA in cells is to express a fluorescently labeled RecA in a WT background. This strategy showed that RecA in *Escherichia coli* initially nucleated at the DSB locus and then extended to dynamic bundle or filament structures (Figure 2A) [101,102]. In these experiments, RecA filaments formed before the rapid movement and pairing of two sisters, indicating that homology search happened along the RecA structures. Further work revealed that RecA filaments functioned by a reduced-dimensionality mechanism (Figure 2B) [102]. An extensive homology search is not required when DSBs occur at stalled DNA replication forks. In this case, RecA formed transient foci instead of bundles or filaments [103]. These observations suggest a diversity of RecA dynamics corresponding to different types of DSBs, describing a context-dependent homology search process.

The second activity of RecA filaments is recruiting factors to promote the homology search and strand invasion reactions. RecN is an SMC-type protein that has been shown to aid the homology search. The RecN protein from *Deinococcus radiodurans* and *E. coli* promotes RecA-mediated D-loop formation *in vitro* [104,105]. Furthermore, RecA filaments in the bacterium *Caulobacter crescentus* show directional movement during the homology search and undergo several pole-to-pole traversals *in vivo*. Without RecN, RecA filaments remain at the damaged sites, cannot move across the cell, and fail to repair DNA [106]. This pole-to-pole movement illustrates that a systematic scan of genomic DNA is a part of the homology search in bacteria (Figure 2C).

In eukaryotes, the direct labeling of Rad51 was achieved in live cells by introducing a fluorescent tag in the least conserved region of *S. cerevisiae* Rad51 N-terminus. This advance allowed for direct visualization of the homology search and described a Rad51 filament that was able to undergo repeated rounds of expansion and contraction while searching for a homologous sequence (Figure 2D) [107]. This contrasted the bacterial search model and allowed for the search of a spherical nucleus. Without the repeated rounds of expansion and contraction, the rigid Rad51 filament would remain stuck in the same place. This would prevent an exhaustive search of the genome. These observations were a major advance in understanding eukaryotic homology search.

This study highlighted that the long-range DNA resection machinery of Exo1 and Sgs1 contributed to the homology search, suggesting long-range search may require longer tracts of ssDNA. Likewise, Srs2 and Rad54 contributed to controlling the occupancy of Rad51 within a filament. Like in bacteria, short-range searches are likely carried out by Rad51 foci, suggesting a common mechanism for RecA/Rad51 mediated homology search.

In summary, physiological mechanisms that govern the homology search appear to occur through the expansion and contraction of RecA/Rad51 filaments or mediated genome scanning. The ability of RecA/Rad51 to transition from local structures to expanded filaments signifies an ability to conduct both local and long-range searches. Future work will need to be directed at understanding how the accessory factors that aid Rad51 contribute to these expansion and contraction-based search mechanisms.

## Conclusion

The observations so far paint an elegant but incomplete picture of the mechanisms governing the homology search. It remains a difficult problem to solve because most strategies designed to probe the question rely on end-point measurements that genetically perturb the structure of Rad51 filaments or D-loops. Further exploration of this subject will be required to complete the picture. Functionally connecting physical observation to genetic observations will require the direct observation of homolog pairing *in vivo* combined with functional mutations in regulating enzymes. Given that most enzymes are now known to show diverse functions and perform in more than one stage of HR, these experiments need to involve specific functional mutants instead of deletions. This will preserve the overall structural integrity of the Rad51 filament while isolating known functions of accessory factors.

## Perspectives

- The dynamic nature of the homology search is critical for understanding the outcomes of HR.
- The current thinking on this topic is that homology search is a dynamic process that RecA/Rad51 regulates through both enzymatic function and recruitment of accessory factors that stimulate the formation and reversal of homology search intermediates.
- Future directions in this area will be to understand how accessory homology search factors contribute to the rate of both long-range and short-range homology searches co-ordinated by Rad51.
- Chromatin remodeling upon DNA damage regulates chromosome movement and homology search efficiency.

## Abbreviations

BIR: break-induced replication
CO: crossover
CTD: C-terminal domain
dHJ: double Holliday junction
DSB: double-strand break
HR: homologous recombination
PSC: presynaptic complex
RPA: replication protein A
SMC: structural maintenance of chromosome
